# A Recipe to Evolve Complex Life Chemically on Earth

**DOI:** 10.3390/genes16101136

**Published:** 2025-09-25

**Authors:** Lei Lei, Zachary Frome Burton

**Affiliations:** 1 School of Biological Sciences, University of New England, Biddeford, ME 04005, USA; llei@une.edu; 2 Department of Biochemistry and Molecular Biology, Michigan State University, East Lansing, MI 48824, USA

**Keywords:** abiogenesis, astrobiology, tRNA, polyglycine, evolution of the genetic code, origin of life, protocells

## Abstract

Sequences of tRNAs are highly patterned in easily identifiable RNA repeats and RNA inverted repeats (stem–loop–stems). Because of patterning, the multi-step evolution of tRNA can be described in remarkable detail. To evolve life on Earth or another planet or the moon requires the evolution of tRNA or a tRNA-like molecule to act as a genetic adapter. To replace tRNA with an alternate or improved genetic adapter is a remarkably challenging problem, indicating strong chemical selection of tRNA precursors in pre-life. The genetic code, translation systems, and first proteins coevolved with tRNAomes (all of the tRNAs of an organism). Because the tRNA sequence can be separated into component parts, a simple pathway for chemical evolution of life and genetic coding can be described in sufficient detail to allow the assembly of a living entity in laboratories.

## 1. Introduction

Because features of complex life emerge with tRNA and tRNAomes, the evolution of tRNA is the central and most essential pathway to evolution of a genetic code and complex life supported by coding [1,2,3,4]. To generate complex life requires a genetic code, which cannot be evolved except by evolving a genetic adapter. The tRNA molecule arose with specialized features that make tRNA difficult to improve or replace. Life on Earth coevolved with tRNA, tRNAomes, and the genetic code. On another planet or the moon, we suggest that life must evolve by a very similar mechanism, utilizing tRNA or a very similar tRNA-like molecule.

Evolution of tRNA, which occurred about 4.2 billion years ago, has been described in detail [1,3]. The original tRNA molecule was generated from GCG, CGC, and UAGCC repeats and stem–loop–stems (CCGGG_CU/NNNAA_CCCGG and UAGCCUAGCCUAGCCUA; N indicates sequences scrambled in coding; _ separates sequence features such as stem–loop–stems; / indicates a U-turn) [1,3]. The pattern is obvious from an analysis of typical tRNA diagrams and sequence logos. The original tRNA molecule evolved from ligation of three 31 nt minihelices of mostly known sequences (GCGGCGG_UAGCCUAGCCUAGCCUA_CCGCCGC and GCGGCGG_CCGGG_CU/NNNAA_CCCGG_CCGCCGC). In this paper, we substitute the glycine anticodon GCC for NNN. We explain this by simplifying and possibly correcting the assignment below. Ligation of 31 nt minihelices was followed by internal deletions of 9 nt within ligated acceptor stems (CCGCCGC_GCGGCGG). The only sequence ambiguities in the pathway (indicated by N) are the bases that have been altered in coding to form tRNAomes. It is possible that the T loop of tRNA was generated from the complement of the anticodon loop minihelix sequence because the complementary sequence is almost identical to the sequence given [1]. ACCA-Gly ligated at the 3′-end allowed the initial tRNA molecule to be utilized to synthesize polyglycine. We suggest that, before the advent of sequence-dependent proteins, polyglycine was a major initial chemical driving force supporting the evolution of living systems. The original print of the tRNA sequence, therefore, was highly patterned and ordered, and shows how the molecule was generated in pre-life. Life evolved around tRNA and the tRNA anticodon loop, explaining why tRNA sequences in living organisms are so highly conserved from pre-life, while interacting systems have become more innovative.

tRNA, tRNAomes, aminoacyl-tRNA synthetases, the first proteins (proteins coevolved with the genetic code), the genetic code, ribosomes, and the first cells coevolved. If the tRNA molecule were not generated early in the process, the complex coevolution could not have advanced. There may be very few or no alternative routes than tRNA to the evolution of life on Earth or on other celestial bodies.

To our knowledge, a recent review that integrates the tRNA sequence, structure, and evolution has not been published. Here, we attempt to construct a straightforward description of the tRNA sequence, structure, and evolution that can be used as a guide to recreate many of the core steps in the emergence of complex life on Earth. Life can be defined in various ways. Here, we refer to complex life as manifested in the first microbial cells, supported by a genetic code. The evolution of tRNA and tRNAomes was a prerequisite to the origin of microbial life.

## 2. Materials and Methods

tRNA sequences were obtained from the genomic tRNA database (gtRNAdb) [5] and the tRNA Gene DataBase Curated by Experts (tRNADB-CE) [6,7,8]. RCSB (Research Collaboratory for Structural Bioinformatics) Protein Data Bank [9,10,11] files were imaged using ChimeraX (version: 1.10.1 (24 July 2025)) [12,13,14].

## 3. Type II tRNA

Type II tRNA has a longer V arm compared to a type I tRNA V loop [1,2]. Otherwise, type II and type I tRNAs are homologous over their entire lengths. The type II V arm was initially 14 nt, generated by ligation of a 3′-acceptor stem (7 nt; initially CCGCCGC) to a 5′-acceptor stem (7 nt; initially GCGGCGG). The type I V loop was processed from an initial type II V arm by a 9 nt deletion (eliminating GCGCGGCGG). The initial sequence of a type I V loop was CCGCC. The type II V arm evolved to form a stem–loop–stem with a characteristic trajectory from the body of the tRNA. Typically, the cognate aminoacyl-tRNA synthetase (AARS; i.e., LeuRS-IA and SerRS-IIA) recognizes the type II V arm by its sequence and/or its trajectory. In Archaea, type II tRNAs (tRNA^Leu^ (5 anticodons) and tRNA^Ser^ (4 anticodons)) utilize the type II V arm rather than the anticodon loop as a major determinant for accurate amino acid addition [2]. The type I V loop was selected to form contacts to other tRNA residues. Type I tRNAs generally utilize the anticodon loop as a major determinant for cognate tRNA recognition (type I tRNA^Ala^ is an exception) and not the shorter type I V loop.

**Figure 1:**
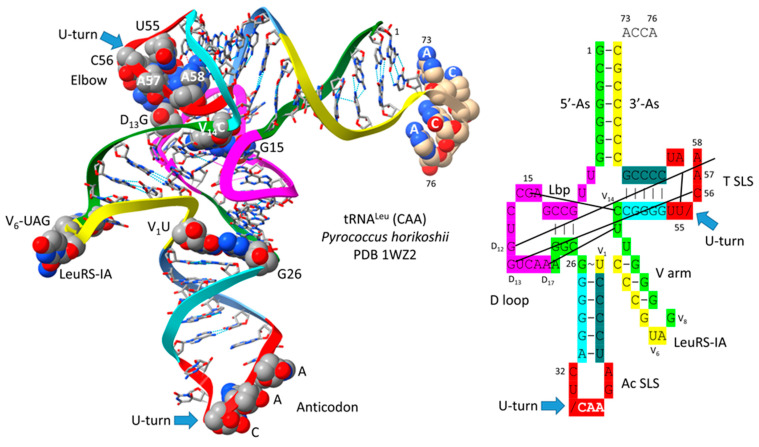
Type II tRNA. A tRNA^Leu^ (CAA) from *P. horikoshii* (an ancient Archaeon) is shown (PDB 1WZ2) [15,16]. The image is colored according to the three 31 nt minihelix theorem for the evolution of tRNA. Consistent colors indicate homologous sequences: green: 5′-acceptor stem (5′-As), 5′-As* (5′-acceptor stem remnant homologous to 5′-As tRNA-3-7), and V_8_–V_14_ of the type II V arm; magenta: the D loop 17 nt minihelix core; yellow: V_1_–V_7_ of the V arm and the 3′-acceptor stem (3′-As); cyan: anticodon and T 5′-stem; red: anticodon loop and T loop; cornflower blue: anticodon and T 3′-stem. 3′-ACCA is uncolored. / indicates a U-turn in the RNA backbone. SLS indicates stem–loop–stem. Lbp indicates the Levitt reverse Watson–Crick base pair between tRNA-15G (D_8_G) and V_14_C. LeuRS-IA binds V_6_-UAG-V_8_ as a determinant for cognate charging of tRNA-76A 2′-O of the ribose ring with leucine.

Figure 1 shows a type II tRNA^Leu^ (CAA) from the ancient Archaeon *Pyrococcus horikoshii*, colored according to the three 31 nt minihelix tRNA evolution theorem [1,3]. Sequences with a common color are homologous to one another. We suggest that the *P. horikoshii* tRNA^Leu^ (CAA) [15,16] is very similar to a tRNA^Leu^ from LUCA (the last universal common (cellular) ancestor) [17,18] because its sequence is very close to the primordial sequence. To the right of the image is a 2-dimensional tRNA schematic diagram with consistent coloring. Historic numbering of tRNAs breaks down in the D loop because of deletions, and in the type II V arm and type I V loop, because type II V arm and type I V loop sequences were misaligned and because of indels (insertions and deletions). Here, we use D loop numbering D_1_ to D_17_. For the type I V loop, we number V_1_ to V_5_. For the type II V arm, we number V_1_ to V_n_ (V arm of n bases). In the absence of indels, V_1_ to V_5_ align for type I V loops and type II V arms. The type I V loop was processed from an early version of the type II V arm by a 9 nt internal deletion within ligated 3′- and 5′-acceptor stems (CCGCCGC_GCGGCGG was shortened to CCGCC with GC_GCGGCGG deleted).

In Figure 1, color coding corresponds to internal tRNA homologies. The 17 nt anticodon and the 17 nt T stem–loop–stems are homologs. Because the complementary sequence of the anticodon stem–loop–stem is almost the same as the forward sequence, the T stem–loop–stem may be derived from the complement of the anticodon stem–loop–stem rather than the direct anticodon stem–loop–stem sequence [1]. The 5′-As* sequence (tRNA-22 to tRNA-26; green) is homologous to tRNA-3 to tRNA-7 of the 5′-As (As for acceptor stem).

The tRNA^Leu^ (CAA) image in Figure 1 is from a co-crystal with the LeuRS-IA AARS charging enzyme [15,16]. The 3′-ACCA is bent down into the LeuRS-IA aminoacylating active site for the addition of leucine (the “hairpin” conformation). LeuRS-IA binding causes some unwinding of the tRNA^Leu^ anticodon loop, although LeuRS-IA does not bind the anticodon loop directly. Because leucine is in a six-codon box in the standard genetic code and utilizes five tRNA^Leu^, LeuRS-IA binds the type II V arm instead of the five different anticodon loops as a determinant for cognate tRNA^Leu^ charging.

**Figure 2:**
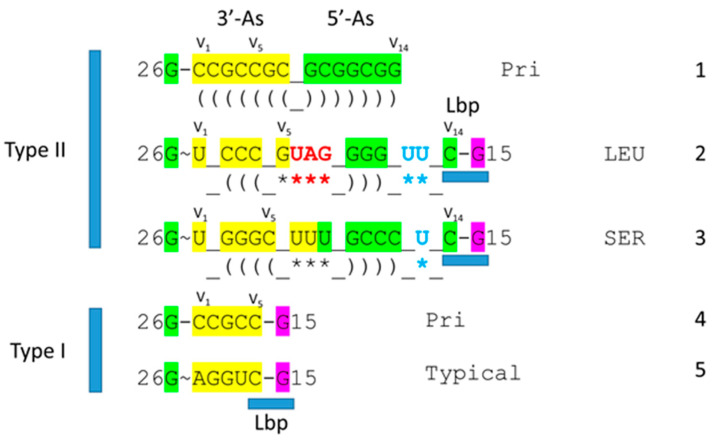
Evolution of type II V arms and type I V loops at the base of the genetic code. Pri indicates the type II (line 1) and type I (line 4) primordial (pre-life) sequences. The type I V loop was processed from the primordial type II V arm sequence. Examples of archaeal tRNA^Leu^ (line 2) and tRNA^Ser^ (line 3) V arm sequences are shown. A typical and common type I V loop sequence from Archaea is shown (line 5). tRNA^Leu^ type II V arm V_6_-UAG-V_8_ (red) binds LeuRS-IA as a determinant for cognate tRNA^Leu^ charging [15,16]. Cyan bases determine the trajectory of the type II V arm from the tRNA body. _ separates stems and loops. (and) (parentheses) indicate stems. * indicates loops. Colors as in other figures.

Figure 2 shows a comparison of type II V arms and type I V loops [1,2]. Type II V arms were derived from ligation of a 7 nt 3′-acceptor stem (CCGCCGC) to a 7 nt 5′-acceptor stem (GCGGCGG) [1,2]. Such a 14 nt sequence could pair along its entire length. The 14 nt V arm sequence evolved to the tRNA^Leu^ and tRNA^Ser^ type II V arms by forming distinct stem–loop–stems with different cognate trajectories from the tRNA body. V_1_U interacts with tRNA-26G, forming a single hydrogen bond. The V arm is utilized as a determinant for cognate Leu and Ser charging at the tRNA 3′-CCA end. The trajectory of the V arm from the tRNA body is determined from the number of unpaired bases between the 3′-V arm stem and the Levitt base CV_n_ (for a V arm of n nucleotides). To specify cognate charging, the trajectory of the V arms is typically distinct for the set of tRNA^Leu^ (five tRNA^Leu^) and the set of tRNA^Ser^ (four tRNA^Ser^) and is a determinant for cognate recognition [2].

Leucine and serine are in six-codon boxes in the standard genetic code. The type II V arm is utilized as a major determinant for cognate leucine and serine charging of tRNAs. In Archaea, the type II V arm is only utilized by tRNA^Leu^ (five tRNA^Leu^) and tRNA^Ser^ (four tRNA^Ser^). In Bacteria, tRNA^Tyr^, tRNA^Leu^, and tRNA^Ser^ utilize type II V arms [2]. In Archaea, almost all tRNA^Leu^ V arms are 14 nt in length, which is the primordial length. Ligating a 7 nt 3′-acceptor stem to a 7 nt 5′-acceptor stem generates a 14 nt sequence (initially CCGCCGC_GCGGCGG) (Figure 2, line 1). The type II tRNA^Leu^ and tRNA^Ser^ V arms evolved to form stem–loop–stems utilized as determinants by LeuRS-IA and SerRS-IIA for cognate amino acid charging at tRNA-76A. The type I V loop was processed from the primordial type II V arm sequence (initially CCGCCGC_GCGGCGG processed to CCGCC) (line 4).

Type II tRNAs and type I tRNAs are homologous over their entire lengths except for the 9 nt deleted segment in the type I tRNA V loop region. Most contacts in type II tRNAs and type I tRNAs are the same. Some core interactions are noted in Figure 1. The “elbow” of tRNA is where the D loop and the T loop interact. D_12_G intercalates between tRNA-57A (sometimes 57G) and tRNA-58A and hydrogen bonds to tRNA-55U, just before the T loop U-turn. D_13_G forms a slightly bent Watson–Crick pair with tRNA-56C. The Levitt reverse Watson–Crick base pair connects tRNA-15G (D_8_G) and V_14_C. A reverse Watson–Crick pair interacts at the same face as a Watson–Crick pair but with one of the bases flipped over. A G=C reverse Watson–Crick pair forms two hydrogen bonds.

## 4. Type I tRNA

**Figure 3:**
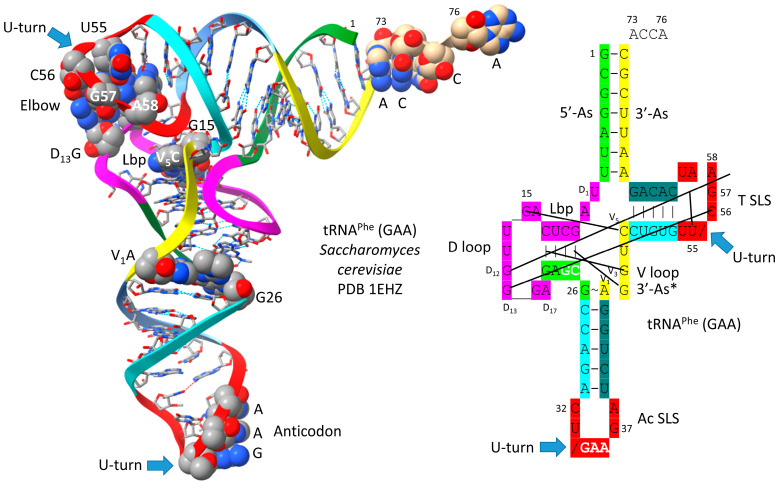
Type I tRNA. A tRNA^Phe^ (GAA) from *S. cerevisiae* is shown (PDB 1EHZ) [19]. Colors and labels are as in Figure 1. The intercalating base D_12_G is mostly obscured in the image on the left. Deleted bases are indicated by _. 3′-As* is homologous to tRNA-66 to 70.

A type I tRNA^Phe^ (GAA) from *Saccharomyces cerevisiae* is shown in Figure 3 [19]. Although this is a eukaryotic tRNA^Phe^, it is very similar in structure and sequence to an archaeal tRNA^Phe^. The PDB 1EHZ structure was selected because of its high resolution and completeness. Also, the tRNA^Phe^ (GAA) is fully modified as in vivo. The discussion above for type II tRNA^Leu^ mostly describes the tRNA^Phe^ (GAA) structure. The 17 nt D loop core sequence (magenta) has 3 nt deleted (underscored positions). The V loop sequence is 5 nt in length (the primordial length), with the typical and common sequence V_1_-AGGUC-V_5_ (Figure 2; line 5). V_1_A interacts with tRNA-26G forming a single hydrogen bond. V_2_G interacts with the D stem. tRNA-10G (D_3_G) pairs with tRNA-25C, and V_2_G-10G-25C form a triplex interaction. Similarly, V_3_G forms a triplex interaction with 22G=13C (D_6_C). V_4_U flips away from the body of the tRNA. The elbow contacts and Levitt reverse Watson–Crick pair (tRNA-15G (D_8_G) binds V_5_C) are as described above for the type II tRNA^Leu^ in Figure 1.

## 5. Was the First tRNA a tRNA^Gly^?

**Figure 4:**
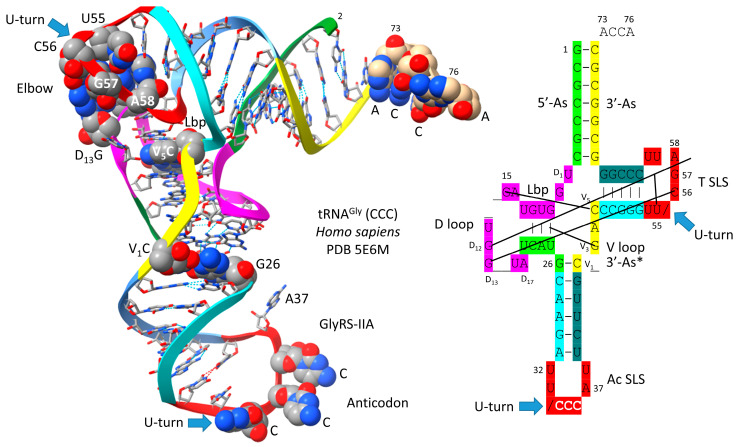
Human tRNA^Gly^ (CCC) from a co-crystal with GlyRS-IIA [20]. D_12_G is mostly obscured in the image on the left. Anticodon loop residues 35-CCA-37 are unwound to interact with GlyRS-IIA as a determinant for cognate tRNA^Gly^ (GCC, UCC, CCC) charging with glycine. 3′-As* is homologous to tRNA-66 to 70. Colors as in other figures.

Figure 4 shows a human tRNA^Gly^ (CCC) [20]. The image was selected as the best available tRNA^Gly^ image with the highest similarity to archaeal tRNA^Gly^. The tRNA^Gly^ (CCC) was taken from a co-crystal with GlyRS-IIA. GlyRS-IIA unwinds the anticodon loop to expose 35-CCA-37 as a determinant for cognate tRNA^Gly^ (CCC) charging with glycine at 3′-ACCA (at the 3′-O of the ribose ring of 76A). From the primordial sequence, 4 nt was deleted from the 17 nt D loop core, as indicated in the schematic. Also, 1 nt (V_2_ or V_3_) was deleted within the type I V loop. V_3_G forms a triplex interaction with D stem residues D_3_G and 25U, as indicated in the schematic.

**Figure 5:**
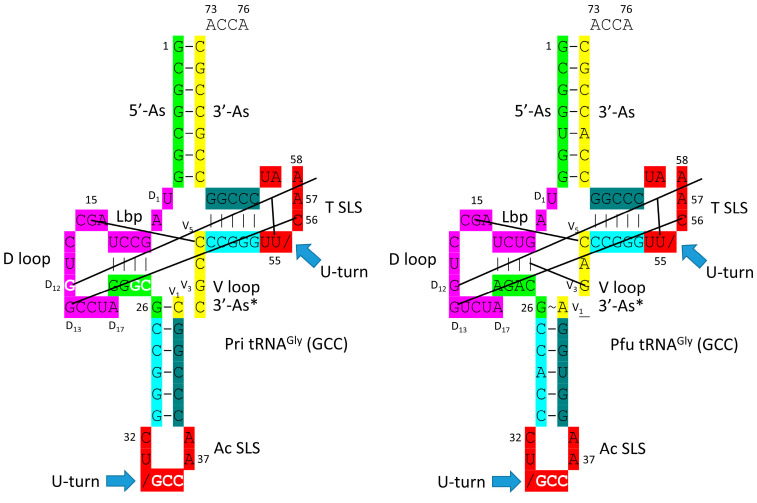
Comparison of tRNA^Pri^ (Pri for primordial) and tRNA^Gly^ (GCC) of *P. furiosus* (Pfu). We suggest that tRNA^Gly^ was the first tRNA, which evolved initially to synthesize polyglycine.

In Figure 5, schematic diagrams of a primordial (Pri) type I tRNA and an archaeal *Pyrococcus furiosus* tRNA^Gly^ (GCC) are shown. We have assigned a GCC anticodon to tRNA^Pri^, as we explain below. The sequences are very similar after ~4.2 billion years, making tRNA^Gly^ derived from an ancient Archaeon a living fossil of the inception of life. It follows that the first tRNAs on Earth were utilized to synthesize polyglycine. In an Archaeon, GlyRS-IIA appears to be the most ancient AARS enzyme. Chemical selection for polyglycine is suggested to have driven the evolution of the first cells. tRNA^Gly^ appears to be the first tRNA from which other tRNAs were derived [21]. In an ancient Archaeon, tRNA^Gly^ is the most similar tRNA to tRNA^Pri^.

**Figure 6:**
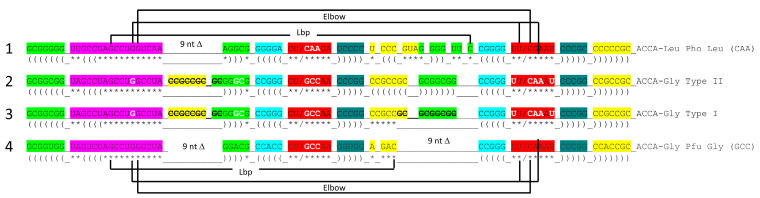
Generation of type II and type I tRNAs at the base of code evolution. Pho: *P. horikoshii* (see Figure 1); Pfu: *P. furiosus* (Figure 4). Levitt base pair (Lbp) and elbow interactions are indicated. Deleted bases are bold with strikethrough. Colors as in other figures.

In Figure 6, evolutionary conservation of the first type II and type I tRNAs is summarized. tRNA^Leu^ (CAA) from *P. horikoshii* is shown in line 1 (see also Figure 1). The primordial type II tRNA sequence from which tRNA^Leu^ and tRNA^Ser^ were derived is shown in line 2. A tRNA^Gly^ (GCC) from *P. furiosus*, which is an ancient Archaeon, is shown in line 4 (Figure 5; right panel).

## 6. Breaking tRNA^Pri^ into Its Component Parts

To generate complex life supported by a genetic code in a laboratory requires the directed evolution of tRNA^Pri^. Because tRNA^Pri^ evolved from RNA repeats (GCG, CGC, and UAGCC) and inverted repeats (CCGGG_CU/NNNAA_CCCGG), this goal appears tractable. The only slight deviation from order in tRNA^Pri^ is 3′-ACCA-Gly, which is a short adapter molecule that was attached to many RNAs during the chemical evolution of life [1]. tRNA^Pri^, therefore, can be broken into its separate parts. Those components can then be separated and combined, and processes can be inferred for transitions that resulted in tRNA^Pri^ evolution. Because tRNA^Pri^ was so highly ordered, its evolution pathway was defined, and intermediates in tRNA evolution were identified. Reproducing transitions between these components would be a major contribution to understanding the evolution of life.

**Figure 7:**
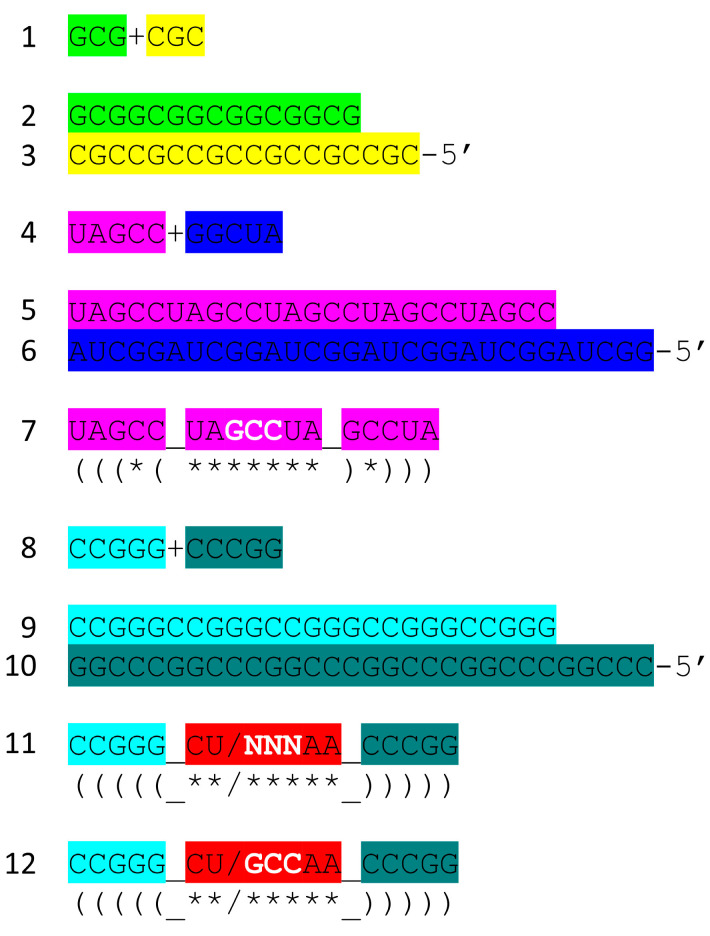
tRNA^Pri^ evolved from RNA repeats and inverted repeats. Colors are as in other figures. See the text for details. We infer that GGCUA repeats (blue) were present as the complement of UAGCC repeats (from which the 17 nt D loop minihelix core of tRNA was derived) (lines 4–7). Lines 8–12 indicate sequences that must be generated to obtain the anticodon and T 17 nt stem–loop–stems. At this time, we do not have a clear idea of how the anticodon and T 7 nt U-turn loops were first generated. It is clear, however, why the 7 nt U-turn loop was selected (see below). Colors as in other figures.

Figure 7 shows the tRNA^Pri^ components that require synthesis. 5′-acceptor stems evolved from GCG repeats. 3′-acceptor stems evolved from complementary CGC repeats. We infer that a chemical mechanism evolved to generate GCG and complementary CGC repeats on pre-life Earth (Figure 7, lines 1–3). The conservation of GCG and CGC repeats in tRNAs implies a complementary replication mechanism on pre-life Earth. Because processive 5′ to 3′ ribozyme complementary replication has been somewhat difficult to reproduce in laboratories, perhaps ligation on a template was the mechanism utilized for initial complementary replication [22,23,24,25,26].

More complex RNA repeats were also synthesized. The 17 nt D loop minihelix core was based on a UAGCC repeat (initially UAGCCUAGCCUAGCCUA) (Figure 7, lines 4–7). Because GCG and CGC repeats are complementary, we infer that GGCUA repeats (blue) were also synthesized on pre-life Earth (lines 4 and 6). We note that a 17 nt UAGCC repeat can fold into a stem–loop–stem that presents a GCC anticodon (line 7). We infer that this molecule could attach 3′-ACCA-Gly for use in polyglycine synthesis using an extended GCG repeat (line 2) as a template. Because the GGCUA repeat (line 6) was not preserved in tRNA sequences, this sequence appears to be extinct now. We infer that many RNA repeats and inverted repeats were present on pre-life Earth, and perhaps only those repeats preserved in tRNA sequences survived the transition to Darwinian selection with the evolution of the first cells.

In addition to the 17 nt UAGCCUAGCCUAGCCUA stem–loop–stem (Figure 7, line 7), an essential 17 nt stem–loop–stem with a 7 nt U-turn loop evolved (Figure 7, lines 8–12) (CCGGG_CU/NNNAA_CCCGG or CCGGG_CU/GCCAA_CCCGG). We argue that the 7 nt U-turn loop (i.e., CU/NNNAA or CU/GCCAA) was the most important innovation in pre-life chemical evolution on Earth. Without this specialized and ribozyme nuclease-resistant loop, tRNA could not have evolved as the genetic adapter. Without a genetic adapter as good or better than tRNA, complex life with coding could not have evolved. By the time of LUCA, the sequence designated NNN was scrambled in the evolution of tRNAomes. If the anticodon sequence was originally GCC, as in the 17 nt UAGCC repeat, and with 3′-ACCA-Gly added at the RNA 3′-end, an extended GCG repeat could be utilized as a template to synthesize polyglycine. We favor the idea that polyglycine was the main selective driver of chemical evolution during pre-life on Earth.

## 7. Chemical Evolution of the First Translation Systems

**Figure 8:**
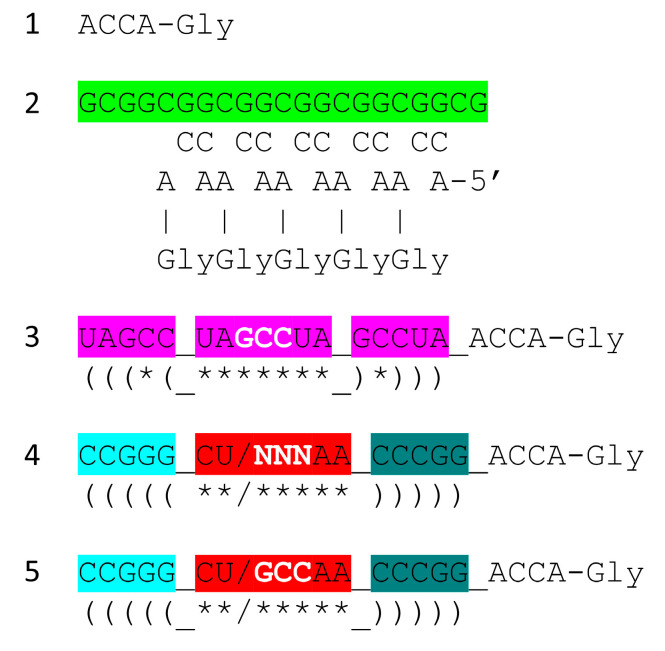
A model for the evolution of the first translation systems. Bringing multiple ACCA-Gly into proximity within an RNA environment should be sufficient to synthesize polyglycine, perhaps driven by wet–dry cycles. Colors as in other figures.

In Figure 8, we show a model for one of the first translation systems, and one that should be capable of synthesis of polyglycine in a laboratory. Through Darwinian selection, the model ought to be capable of extension to evolve a fairly modern form of the ribosome. We propose that ACCA-Gly found in most tRNAs was the most primitive adapter molecule [1]. An extended GCG repeat includes many iterations of the sequence CGGC which can pair with ACCA-Gly to bring many ACCA-Gly into proximity within an RNA environment (Figure 8, lines 1 and 2). The synthesis of polyglycine and polypeptides involves dehydration. The peptidyl-transferase center of the modern ribosome can be viewed as a dehydration and orientation center to facilitate tRNA-linked peptide bond formations at the A and P sites (A for aminoacyl and P for peptidyl sites). A tangled GCG repeat forms a dehydrating environment because polar RNA binds water. Also, wet–dry cycles can promote dehydration for glycine polymerization. We suggest that the system shown schematically in line 2 can be utilized with wet–dry cycles to form polyglycine using published procedures [27].

A second-generation polyglycine synthesis system is also indicated (Figure 8, lines 3–5). We hypothesize that ACCA-Gly was ligated to many RNAs during pre-life. Consequently, 17 nt stem–loop–stems with 3′-ACCA-Gly, combined with an extended GCG repeat (a primitive pre-ribosome), with wet–dry cycles, should be capable of synthesizing polyglycine. From an analysis of tRNA evolution and sequence, we see no reason to require the synthesis of more complex polypeptides than polyglycine prior to the evolution of tRNA. This does not mean that more complex polypeptides than polyglycine were not present, but they likely would have been synthesized using other mechanisms. Lines 3–5 indicate the evolution of stem–loop–stem snap-back primers for complementary replication, which may have initially involved assembly and ligations of short RNAs on a complementary RNA template framed by snap-back primers. Such a mechanism requires a ribozyme ligase and ribozyme endonucleases to excise products.

## 8. Evolution of Third- and Fourth-Generation Polyglycine Synthesis Systems

**Figure 9:**
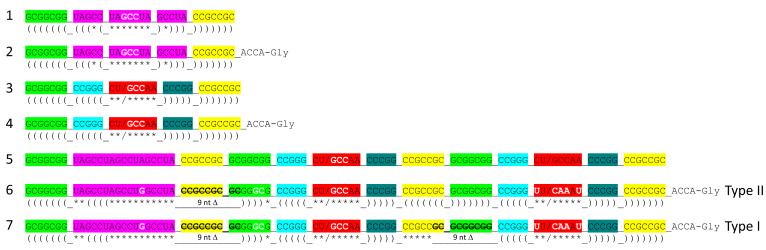
Type II and type I tRNAs evolved chemically by ligation of three 31 nt minihelices followed by internal 9 nt deletion(s) within ligated acceptor stems. Deleted bases are indicated by strikethrough and bold type. Colors and notations are as in other figures. Only a few sequences were systematically changed in forming tRNA from minihelices (i.e., white letters). Colors as in other figures.

Figure 9 indicates further evolution of polyglycine synthesis systems, 31 nt minihelices (lines 1–4), and tRNAs (lines 5–7). As previously described, tRNA was generated by ligation of three 31 nt minihelices: one 31 nt D loop minihelix (line 1) and two 31 nt anticodon stem–loop–stem minihelices (line 3) [1,3,28]. Thus, a 93 nt tRNA precursor was formed as a segment of a replication intermediate for 31 nt minihelices. We imagine the 93 nt tRNA precursor as part of a much larger molecule that includes snap-back primers and the complementary strand. The 93 nt precursor was then processed by a single 9 nt internal deletion within ligated 3′- and 5′-acceptor stems to form a primordial type II tRNA (line 6). To form type I tRNA, an additional internal 9 nt deletion occurred within the V loop region (line 7). Remarkably, the two internal 9 nt deletions to form type I tRNAs are identical on complementary strands, once again indicating complementary replication in the pre-life world.

The mechanism proposed for the synthesis of the first tRNAs (Figure 9, lines 1–7) can be extended to more complex molecules, such as rRNAs and first proteins [1]. Very clearly large complex RNAs could be generated by ligation of multiple RNAs. The 93 nt precursor from which tRNA was derived is thought to be part of a much larger, circular RNA molecule capped with the ligation of stem–loop–stems (i.e., Figure 7, lines 7, 11, and 12; and Figure 9, lines 1 and 3) before folding and excision of tRNAs. Once translation systems evolved, the translation of ligated RNAs would generate some of the first complex proteins. There is little reason to assume that early RNAs and proteins were necessarily simple molecules that assumed more complex forms later in evolution. In pre-life, many RNAs and the first proteins that coevolved with the genetic code were long, varied, and complex.

## 9. Alternate Genetic Adapters

**Figure 10:**
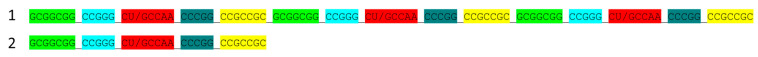
Three ligated anticodon stem–loop–stem 31 nt minihelices are expected to fold and be processed to three 31 nt anticodon stem–loop–stem minihelices, emphasizing the need for the 5′-D loop 31 nt minihelix to generate tRNA (Figure 9; lines 5–7). Colors as in other figures.

Somewhat surprisingly, there does not appear to be a large number of alternatives to the genetic adapter tRNA that evolved on planet Earth. Part of the problem is illustrated in Figure 10. If the D loop minihelix were to be replaced at the 5′-end of the tRNA precursor by a third anticodon loop minihelix, folding into a tRNA becomes much more unlikely. The greater flexibility of the D loop 17 nt minihelix core, compared to the stiffness of the anticodon stem–loop–stem minihelix, allows for tRNA folding. Ligation of three anticodon stem–loop–stem minihelices (Figure 10, line 1) would be expected to be processed to three anticodon stem–loop–stem 31 nt minihelices (line 2) because of their more stable folding (compare to Figure 9, lines 5–7). These claims can be tested computationally and by experimentation. In evolution, many alternate adapter folds and sequences may have been tested against the pathway that produced type II and type I tRNAs (Figure 9, lines 5–7). The mechanisms that were chemically selected were the fastest mechanisms that resulted in the most successful adapter molecule.

Life as we know it on Earth evolved chemically using the RNA adapter tRNA in an aqueous environment. We know of no other chemistries than aqueous chemistry and RNA chemistry that would have been likely to evolve as enabling a genetic adapter as tRNA. We can imagine a sequence substitution for the 17 nt D loop minihelix core, but that substitution likely could not be a 17 nt anticodon stem–loop–stem (compare Figure 9 and Figure 10). tRNA was generated from RNA repeats and inverted repeats that, apparently, were generated accurately on pre-life Earth. Stem–loop–stems chemically evolved, perhaps to cap linear RNAs for accurate complementary replication via ligation (a ribozyme ligase) or to initiate accurate processive replication. Replacing the 7 nt U-turn loop within the anticodon and T stem–loop–stems also appears problematic. The 7 nt U-turn loop is a compact loop that projects a 3 nt anticodon. The 7 nt U-turn loop, furthermore, is expected to have resisted attack by ribozyme nucleases on pre-life Earth. The tight tRNA anticodon loop (see Figure 3), therefore, appears to have been chemically selected versus competing loops. Also, 31 nt minihelices have longer stems than tRNAs (compare Figure 9, lines 1–4 with lines 5–7). Folding into the more complex tRNA, therefore, may have had advantages compared to minihelices. Apparently, tRNAs were easier to melt and replicate on pre-life Earth than minihelices.

## 10. The Anticodon Loop as Essential Intellectual Property to Evolve Life on Earth

**Figure 11:**
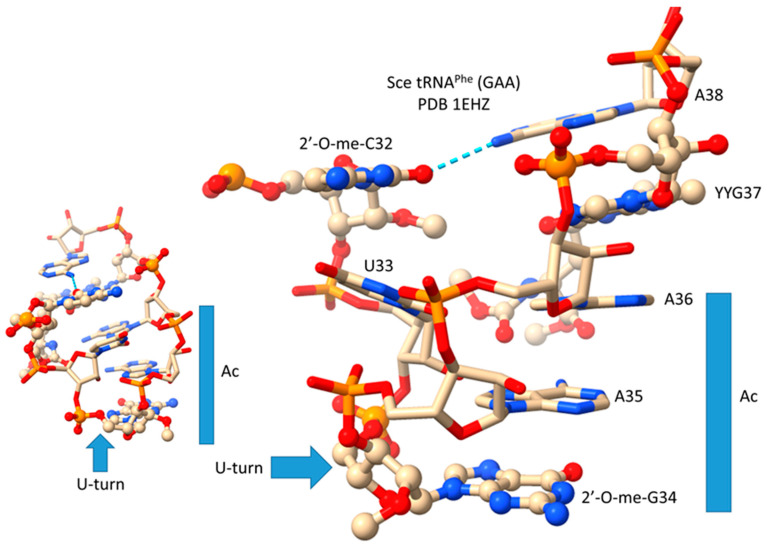
The tRNA anticodon loop. Unmodified bases are in stick representation. Modified bases are in ball and stick representation. Sce for *S. cerevisiae*. YYG for wybutosine (a G modification). Ac for anticodon. Colors: beige: C; blue: N; red: O; orange: P. The view on the left indicates the U-shaped geometry of the U-turn.

In Figure 11, we show the anticodon loop of *S. cerevisiae* tRNA^Phe^ (GAA) (see also Figure 3) [19]. We argue that the compact 7 nt U-turn anticodon loop was a necessary intellectual property to evolve life on planet Earth. Any attempt to substitute the loop with an RNA loop of another length or alternate sequence would probably be unsuccessful in evolving a code. The U-turn is a U-shaped turn in the anticodon loop backbone. A U-turn loop was necessary to form the tight and compact loop to resist ribozyme endonucleases in the pre-life world. The U-turn also projects three nucleotides to form the anticodon. Initially, both tRNA-34 and tRNA-36 may have had wobble positions [1,3,29]. Wobbling at tRNA-36 was suppressed, in part, by modification of tRNA-37. In the tRNA^Phe^ (GAA) shown, tRNA-37 is modified to YYG (wybutosine; a G modification). At the base of code evolution, reading anticodon tRNA-36A also required a tRNA-37G modification (originally, 37m^1^G). To read tRNA-36U required a tRNA-37A modification (originally, 37t^6^A). With unmodified wobble U, tRNA-34U reads mRNA codon 3A, 3G, 3C, and 3U. This is referred to as “superwobbling” and is utilized in mitochondria in 4-codon boxes to shrink the size of the organelle tRNAome [29,30,31]. To read tRNA-34U in the standard code, therefore, U must be modified to restrict its reading to mRNA wobble 3A and 3G. A first protein, termed elongator Elp3, evolved along with the genetic code to support the use of tRNA-34U and to restrict its reading. tRNA-34A was not utilized at the base of code evolution. To generate the first cells, numerous first proteins coevolved with tRNAomes and the genetic code. Because 2′-O-me-C32 and 38A interact (a reverse Hoogsteen interaction), these bases stack with the anticodon stem. Wobbling at tRNA-36 was suppressed, but wobbling at tRNA-34 could not be suppressed in the same way. For one thing, tRNA-33U is on the other side of the U-turn from the anticodon, so modification of tRNA-33U would not alter the reading of tRNA-34. The anticodon loop has specialized properties, modifications, and characteristics that could not easily be substituted by an alternate RNA loop.

## 11. Determinants on tRNA for Cognate Aminoacyl-tRNA Synthetase Recognition

tRNAomes coevolved with the first proteins, aminoacyl-tRNA synthetases (AARSs), which charge tRNAs with their cognate amino acids. AARS enzymes are of two classes: class I and class II. Class II AARSs (i.e., GlyRS-IIA) appear to be more ancient than class I AARSs (i.e., ValRS-IA and LeuRS-IA). Surprisingly, class II and class I AARS enzymes, which have incompatible folds, are homologs by sequence [1,4]. Apparently, class I AARS enzymes were derived from class II AARS by the addition of an N-terminal segment that redirects the fold of class I AARS. We recognize that these data are inconsistent with other models for AARS evolution (i.e., the “urzyme” hypothesis) [32,33,34].

Most tRNAs are type I. Because only a limited set of V arm trajectories are allowed, only a small number of tRNAs can be type II [2]. In Archaea, only tRNA^Leu^ and tRNA^Ser^ are type II. Leucine and serine are in six-codon boxes in the standard genetic code. Having five tRNA^Leu^ and four tRNA^Ser^ presented a problem for cognate tRNA charging utilizing the anticodon loop as an AARS determinant, as is utilized for most tRNAs. tRNA^Leu^ and tRNA^Ser^, therefore, present distinct type II V arms as a major determinant for accurate charging by LeuRS-IA (Figure 1) and SerRS-IIA [2]. Arginine is also within a six-codon sector of the code, but tRNA^Arg^ is a type I tRNA (five tRNAs). ArgRS-IA substantially unwinds the anticodon loop to expose additional bases for cognate tRNA^Arg^ recognition [35]. GlyRS-IIA unwinds the tRNA^Gly^ anticodon loop (three tRNAs) to expose tRNA-35-CCA-37 for recognition (Figure 4) [20]. Apparently, two strategies (type II V arm (Leu and Ser) and unwound anticodon loop (Arg)) were necessary to support three amino acids in six-codon boxes at the base of code evolution [1,2]. Additional determinants are also utilized for cognate amino acid charging including the following: (1) tRNA-73 is the discriminator base, which can be A, G, U or C (initially A); (2) the acceptor stem; (3) the anticodon loop (for all amino acids except alanine, leucine, and serine); (4) the type II V arm (for leucine and serine in Archaea); and (5) the elbow [36,37].

## 12. Dirty Polyglycine and Emulsification at the Origin of Life

When we consider polyglycine to emulsify pre-cell chemistries, we consider “dirty” polyglycine [1]. Polyglycine, therefore, was part of a background of complementary chemistries. Polyglycine can be modified in many ways on pre-life Earth to increase its length, cross-linking, hydrophilicity, and charge. Many such modifications would potentially render polyglycine a better emulsifier of pre-life chemistry. We suggest that polyglycine be tested for its potential reactivity on pre-life Earth and for its promotion of protocell to cell transitions.

## 13. Ribozymes and RNA in Pre-Life

Objections to an RNA world include the possible instability of RNA and some limited capacities of ribozymes to catalyze necessary reactions. RNA that is modified at the 2′-O of the ribose ring, however, is as stable as DNA to base hydrolysis [27]. For instance, 2′-O-methyl single-stranded RNAs and ribozymes are stabilized. RNA modifications must be more ancient than the genetic code because multiple tRNA modifications (i.e., Elp3 modification of 34U, 37m^1^G, 37t^6^A, 34C→agmatidine, 2′-O-meC) were necessary to generate the code [1,3]. Here, we hypothesize a complex mod-RNA–amino acid–protein–metabolism world [27] (mod-RNA for modified RNA). Our view is supported by analysis of tRNA, tRNAome, and genetic code evolution.

## 14. The Three 31 nt Minihelix tRNA Evolution Theorem

There are no theorems (proven models) in biology, but the three 31 nt minihelix tRNA evolution theorem is very close to a proven model. If there is a rational objection to the theorem, we are not aware of it. Other tRNA evolution models have been proposed, but none can be correct [38,39,40,41,42,43,44,45,46,47,48,49,50,51,52]. No convergent or accretion model can be correct because, at the origin of tRNAomes, all tRNAs are homologous along their entire length. Only a divergent model can be adequate for tRNA evolution. tRNAs evolved from a 93 nt precursor that was processed differentially to generate type II and type I tRNAs. All tRNAs in the tRNAsphere radiated from these forms. No other model can account for internal tRNA homologies. No other model can account for the RNA 3 nt (GCG and CGC) and 5 nt (UAGCC) repeats in tRNA or the conserved inverted repeats (initially ~CCGGG_CU/GCCAA_CCCGG; anticodon and T stem–loop–stems). It appears that tRNA is the most strongly conserved sequence for the pre-life to complex life transition. As such, the tRNA sequence provides a powerful gateway to understand the transition on Earth to complex life supported by coding. The three 31 nt tRNA evolution theorem is strongly supported by statistical analyses [21,53,54], and most of its features can readily be confirmed by inspection of the conserved sequence. We support universal acceptance of the three 31 nt minihelix tRNA evolution theorem. Without this acceptance, it is unclear that the pre-life to life transition with genetic coding can be understood.

## 15. Discussion

tRNA appears to be the most highly conserved sequence from pre-life. rRNA is also highly conserved [55], but not as highly conserved as tRNA. Remarkably, the original print of the tRNA sequence has been elucidated, with the exception of those bases scrambled for coding. tRNA was generated from RNA repeats and inverted repeats of known sequences. ACCA-Gly was the primitive adapter molecule. tRNA appears to have evolved initially to synthesize polyglycine. We hypothesize that polyglycine was selected chemically in pre-life for at least two reasons: (1) polyglycine emulsified pre-cellular components to enhance pre-life chemistry; and (2) polyglycine helped form the first protocells and cells [1]. After polyglycine, the genetic code evolved to synthesize GADV polymers [56,57]. At an 8-amino acid stage, the code may have been GADVLSER [1,4,29]. Amino acid-linked chemistry can generate D→N, E→Q and S→C to generate an 11-amino acid stage of code evolution [1,4,29,58]. Suppression of tRNA-36 wobbling allowed the code to expand to 20 amino acids and stops. Fidelity mechanisms froze the code. Because the type II tRNA V arm is a determinant for cognate tRNA charging, only a small set of type II tRNAs can be utilized (in Archaea, tRNA^Leu^ and tRNA^Ser^) [2]. We suggest that, originally, tRNAs were a mixture of type I and type II that were sorted later in evolution, with most tRNAs selected to be type I.

We have taken a top-down, sequence-based approach to the origin of life. A bottom-up approach would be to reproduce pre-life chemistry in a laboratory [59]. When top-down strategies meet bottom-up approaches, an adequate understanding of the pre-life to life transition should emerge. We were surprised at how powerful the top-down strategy proved to be. We were surprised that tRNA sequences were so highly ordered and relayed such a coherent history of the pre-life to life transition on Earth.

tRNA evolution, structure, and function unify biochemistry, coding, and genetics. Incorporating the evolution of tRNA and its relation to the evolution of coding into instruction will improve science education. For tRNA databases, we advocate for a version of our presentation shown here to further advance the core importance of tRNA and coding evolution at the inception of biology.

## Data Availability

No new data were created or analyzed in this study. Data sharing is not applicable to this article.

## Abbreviations

The following abbreviations are used in this manuscript:

AARS	Aminoacyl-tRNA synthetase (ligase)
LUCA	Last Universal Common (Cellular) Ancestor
Elp3	Elongator Protein 3
Indel	Insertions and Deletions
Ac	Anticodon
As	Acceptor stem
SLS	Stem–loop–stem
